# Influence on disease spread dynamics of herd characteristics in a structured livestock industry

**DOI:** 10.1098/rsif.2011.0625

**Published:** 2011-11-23

**Authors:** Tom Lindström, Susanna Sternberg Lewerin, Uno Wennergren

**Affiliations:** 1 IFM Theory and Modelling, Linköping University, Linköping 581 83Sweden; 2 Department of Disease Control and Epidemiology, SVA, National Veterinary Institute, Uppsala 751 89Sweden

**Keywords:** disease spread modelling, heterogeneous contact structure, animal movement

## Abstract

Studies of between-herd contacts may provide important insight to disease transmission dynamics. By comparing the result from models with different levels of detail in the description of animal movement, we studied how factors influence the final epidemic size as well as the dynamic behaviour of an outbreak. We investigated the effect of contact heterogeneity of pig herds in Sweden due to herd size, between-herd distance and production type. Our comparative study suggests that the production-type structure is the most influential factor. Hence, our results imply that production type is the most important factor to obtain valid data for and include when modelling and analysing this system. The study also revealed that all included factors reduce the final epidemic size and also have yet more diverse effects on initial rate of disease spread. This implies that a large set of factors ought to be included to assess relevant predictions when modelling disease spread between herds. Furthermore, our results show that a more detailed model changes predictions regarding the variability in the outbreak dynamics and conclude that this is an important factor to consider in risk assessment.

## 1. Introduction

With the aim of providing deeper understanding of the dynamics of livestock disease, researchers have recently focused much attention on the contact pattern between herds [1–10]. Such analysis may be used for predictions about disease transmission [11] as well as estimation of the effect of a changed contact pattern [12]. This study focuses on animal movements. Different diseases spread with different vectors, but movements of animals between herds are generally considered to be a major risk factor for livestock disease transmission [13].

Theoretical considerations may assume a homogeneous contact pattern with equal probabilities of infections between all herds, known as mass action mixing (MAM), but more realistic models need to include mixing heterogeneities. Perhaps most obvious, the number of contacts may be expected to differ between herds [5,14]. Infected herds with many contacts may rapidly infect a large number of other herds, and thereby function as ‘super spreaders’ [15]. Outbreaks in systems with super spreaders are characterized by heterogeneous transmission dynamics [16]. In other systems, where contacts may be considered undirected, such as, for example, sexually transmitted diseases, it may further be expected that the presence of actors with many contacts will result in a rapid increase in number of infections [17]. The contact structure of animal movements may however be considered highly directed and a herd that sends many animals to other herds may not necessarily receive many [4]. Also, a clustered contact structure is known to influence the dynamic of disease transmission [18]. If infectious contacts occur more frequently within a group of herds, the reproductive ratio generally decreases more rapidly with decreasing number of susceptibles. Assuming that a herd can only be infected once during a defined period of interest implies that reoccurring contacts between two herds will not generate additional disease transmissions. Also, the effect of clustering may be illustrated by how it is usually quantified in network studies, i.e. clustering coefficient, which measures the frequency of ‘triplets’ in the contact network [18].

In this study, we focus on three factors at herd and between-herd levels that may cause heterogeneous contact patterns. First, clustering is typically the effect of a strong spatial component in the contact pattern. Consequently, if contacts are more likely to occur at shorter distance, we may expect a more rapid decrease in the number of new infections owing to depletion of local susceptible herds. Secondly, a large herd may be expected to have more contacts and may thereby be more susceptible to infections as well as potentially infecting many other herds. Thirdly, the production type of a herd is expected to influence both the number of contacts and which herds that will be part of these contacts. Heterogeneity due to different production types is therefore expected to result in heterogeneous disease transmission as well as clustering patterns in the contact structure. In addition, the number of movements from herds of one type to another may be very different from the number of reversed movements [4,5].

Lindström *et al*. [5] presented a model that estimated the probability of movements of pigs between herds based on herd sizes, production types and between-herd distances. In this study we test how inclusion of these aspects is expected to change the dynamics of disease transmission via animal movements. We expect that important characteristics may be missed if these aspects are disregarded in modelling between-herd contacts. Our aim is to investigate the effect of including detailed herd data on the expected dynamics of disease transmission via animal movements. We thereby address the importance of collecting such data.

## 2. Transmission and mass action mixing

In this study, we study infections at the herd level. We follow the assumptions of SIR models, where a herd is considered to be either susceptible (*S*), infected (*I*) or recovered (*R*). A herd is defined as a group of animals that mix homogenously and spread of disease occurs at a much faster rate within than between herds. We further assume that recovered herds remain in state *R* for the time of the outbreak (hence become immune) and no new herds are added during the time of an outbreak. If all herds are equal and have equal probability of contacts, disease transmission could be modelled as an MAM process. In this study, we are not interested in the dynamics of any specific disease, but rather the influence of the observed contact structure, and we therefore want to make as few assumptions as possible about the disease. In order to observe relevant dynamics, we do however need to make some assumptions about the recovery of infected herds. We formulate a discrete model where each step consists of one movement (the source and destination herds are given probabilistically as described later) which results in a new infection if the source herd is infected and the destination herd is susceptible. Hence, we assume that there is a constant rate of movements (which is generally a fair assumption on a seasonal time scale for the considered pig movements [2]). Note that we are not formulating the model in terms of days; rather in terms of movements, and our discrete step length is actually 0.43 h (the analysis of Lindström *et al*. [5] consisted of 20 231 movements over 365 days and hence 1 day = 55.4 movements). However, to make the results transparent, we present our result using the relevant units (days or weeks). We further assume that all infected herds have a constant recovery probability *r* for each step of the model. Defining *I*_*t*_ as the current number of infected herds, the expected number of herds remaining infected is (1 − *r*)*I*_*t*_ and with the assumption of the MAM, the probability of the source farm being infected is ((1 − *r*)*I*_*t*_)/*N*, where *N* is the total number of herds, and the probability of the destination herd being susceptible is given by *S*_*t*_/*N* where *S*_*t*_ is the current number of susceptible herds. Hence, the expected number of susceptible, infected, and recovered (*R*) herds at time *t* + 1 is given by2.1
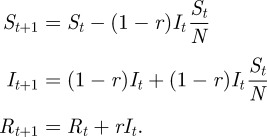
If a disease is initiated with one infected herd at *t* = 0, then *I*_0_ = 1, *S*_0_ = *N* − 1 and initial increase of infected herds is expected if *I*_1_ = (1 − *r*_c_)(1 + 1/*N*) > 1, or rewritten2.2
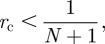
where *r*_c_ is the critical value for when an initial increase in the number of infected herds is expected. In the analysis of pig movements in Lindström *et al*. [5], 3084 herds were analysed. With *N* = 3084 in equation (2.2), *r*_c_ = 3.24 × 10^−4^. The expected time that a herd remains infected, *g*, is given as *g* = 1/*λ*, where *λ* = − log(1 − *r*). In order to calculate *g*_c_, the critical expected time a herd must remain infected for an expected initial increase in the number of infected herds, we set *r* = *r*_c_ and by the assumption of 55.4 daily movements we estimate *g*_c_ at 55.7 days.

## 3. The pig farming industry of sweden

**Figure 1.**
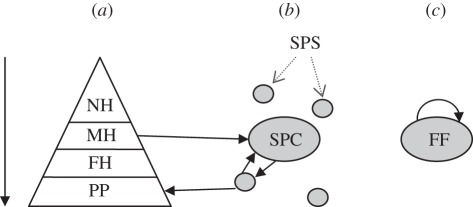
Schematic of the production types with solid arrows showing the main routes of contacts. (*a*) The breeding pyramid is made up of nucleus herds (NH), multiplying herds (MH), piglet producers (PP) and fattening herds (FH). Animal movements occur mainly downwards in the pyramid. (*b*) A sow pool system consist of a sow pool centre (SPC) and several sow pool satellites (SPS). Sows are inseminated at the SPC and sows are moved to SPS for farrowing and are subsequently moved back to the centre. The sow pool system relates to the breeding pyramid such that the SPC mainly obtains gilts from an MH and piglets are moved from SPS to FH for fattening. (*c*) Farrow-to-finish (FF) herds have the whole chain of production integrated at the farm and hence have few contacts with other herds.

The pig industry has a pyramidal structure with transports predominantly going downward in the system; from nucleus (breeder) herds to multiplying (gilt producing) herds, from multiplying herds to piglet producing herds and farrow-to-finish herds, and finally from piglet producing herds to fattening herds (figure 1). Farrow-to-finish herds and fattening herds mainly send animals to slaughterhouses. An exception from the general movement pattern is the sow pool system, where the sows are covered in the central unit and they farrow in the satellite herds. Satellite herds can be either piglet producing or farrow-to-finish herds. The sows are regularly moved back and forth between the central unit and the satellite herds. In Lindström *et al*. [5], it was shown that sow pool centres, nucleus herds and multiplying herds are expected to transmit a disease to a large number of herds, based on the contact structure of animal movements. It was also shown that sow pool centres likely to generate transmission locally, while many long-distance movements are expected from multiplying herds, which may rapidly spread diseases to distant areas. The data used contained 36 herds reported as sow pool centres, 245 as sow pool satellites, 720 as farrow-to-finish, 63 as nucleus herds, 1249 as piglet producers, 88 as multiplying herds and 1147 as fattening herds. Further, 233 herds did not contain information on production types. Some herds had reported more than one production type and in total there were 3084 herds included in the analysis.

## 4. The contact model

In Lindström *et al*. [5], a model was proposed where the probability of a movement between two herds depends on production type, between-herd distance and herd sizes. Parameters were estimated using a hierarchical Bayesian approach, implementing Markov Chain Monte Carlo (MCMC; see Gamerman & Lopez [19] for details on MCMC techniques). The basic concept of the model is presented in this section. But, it is beyond the scope of this paper to give a full description of the model and the parameter estimations involved, and readers are referred to the original paper [5] for further details.

It is assumed that the probability of a movement from herd *s* to *d*, *P*(*d,s*|***θ***), where ***θ*** refers to a set of parameters yet to be specified, can be written as4.1
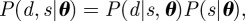
meaning that the destination herd is conditional on the source herd. Herds may have more than one production type, and the model was formulated as a mixture model. Probability of movements between herds were given conditional on the probability of a movement between production types *I* and *J*, referring to the production types of the source and destination herd, respectively. Schematically, the model is written as4.2
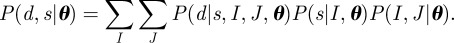
This should be interpreted such that the probability *P*(*d,s*|***θ***) is given as the probability of *d* conditional on *s* and production types *I*, *J* as *P*(*d*|*s,I,J*,***θ***), and the probability of source herd *P*(*s*|*I*,***θ***) is conditional on the production-type *I*. The distribution *P*(*I,J*|***θ***) is defined such that 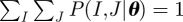 and is given as4.3
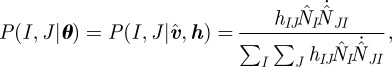
where 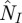 is given as 
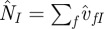
 is the amount of herds registered with production-type *I*, taken into account that a herd is a fraction 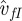 of production-type *I*. Similarly, 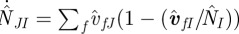 is the amount of herds registered with production-type *J*, but adjusted to exclude the probability of the destination herd being the same as the source herd. For modelling of a system with *K* production types, ***h*** is a matrix of dimensions *K* × *K* and is defined as 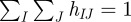, and *h*_*IJ*_ is a measurement of how common movements between production types *I* and *J* are relative to the abundance of the types. In our model, we included the seven production types described in §3, and in addition used a pseudo-type for farms that had not reported a production type, giving *K* = 8. If *h*_*IJ*_ = 1/*K*^2^ for all *IJ*, then movements between all production types are equally probable.

The probability *P*(*s*|*I*,***θ***) was assumed to be dependent on the production-type *I* and herd size. The latter was given by the maximum capacity, which is reported separately for fattening pigs and sows. This was modelled as4.4
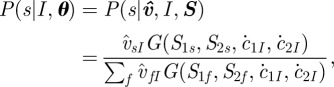
where *S*_1__*s*_ and *S*_2__*s*_ are the maximum capacity of fattening pigs and sows, respectively, reported for herd *s*, and ***S*** refers to the maximum capacity of both demographic groups on all herds. The parameters 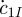 and 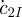 determine how probability of movements depends on *S*_1_ and *S*_2_, respectively, for production-type *I*. The probability of movements was assumed to depend on herd size with a power-law relationship and *G* was given as4.5
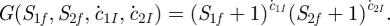
The use of *S*_*uf*_ + 1 (*u* = 1, 2) rather than simply *S*_*uf*_ was used to avoid zero probability of movements from herds with maximum capacity reported as zero for any of the demographic groups. If 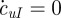, then there is no relationship between maximum capacity of demographic group *u* and the probability of movements starting at a herd for production-type *I*.

The probability of the destination herd *d* of a movement was assumed to depend on both herd size of *d* and the distance between herds *s* and *d*, *D*_*sd*_, and given as4.6
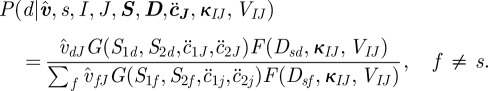
Here, *c̈*_1__*J*_ and *c̈*_2__*J*_ are equivalents of 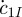 and 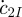, respectively, but used for modelling of the probability of the *destination* herd depending on herd sizes. Consequently, *c̈*_*uJ*_ = 0 (*u* = 1, 2) means that there is no relationship between maximum capacity of demographic group *u* and the probability of movements starting at a herd for production-type *J*. The function *F*(*D*_*sd*_, *κ*_*IJ*_, *ν*_*IJ*_) is a spatial kernel function and describes how probability of movements decreases with distance between herds,4.7
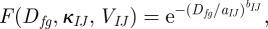
where *κ*_*IJ*_ and *V*_*IJ*_ are the kernel kurtosis and variance, respectively, as4.8
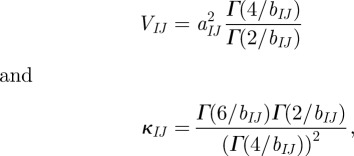
where *Γ* is the gamma function while the parameters *b* and *a* regulate *κ*_*IJ*_ and *V*_*IJ*_ of the spatial kernel (for more details see [20]). In equation (4.6), distance dependence is incorporated by normalizing over all possible destination farms and kurtosis is expected to be of less importance compared with the variance, independent of the spatial arrangement of farms [21].

## 5. Reduced models and simulations

In order to test the effect of the factors included in the contact model on outbreak dynamics, we simulated disease transmission via animal movements with different models. Each replicate was seeded with a single, randomly picked index herd and a herd was assumed to instantly become infected if a movement was simulated from infected herd A to susceptible herd B, and any subsequent movement from B to a susceptible herd was assumed to result in a new infected herd. The mean expected time a herd remains infected (and infective) was set to *g* = 91 days (three months). This generation time was chosen because it is larger than *g*_c_ and hence at least for *M*_MAM_ results in an initial increase of infected herds, yet it is small enough to avoid outbreaks where all herds are infected. Hence it allows for observing relevant aspects of the dynamics. Thousand replicates were simulated with each model and the 3084 herds included in the analysis of Lindström *et al*. [5] were used as infective units in the simulations.

If nothing else is stated, all models were parametrized by random draws (one draw for every replicate) from the full posterior distribution estimated with the MCMC as given in Lindström *et al*. [5] and schematically described in §4. The following five models were used:

— *M*_full_: the full model as described in §4 and [5].
— *M*_1_: by setting *F* (*D*_sd_, *κ*_*IJ*_, *V*_*IJ*_) = 1, distance dependence is removed. Hence, probabilities of contacts depend only on production types and herd sizes.
— *M*_2_: here *h*_*IJ*_ = 1/*K*^2^ for all *I*, *J*, thereby removing the production-type structure of the contact pattern.
— *M*_3_: the probability of contacts depending on herd size was removed by setting 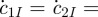 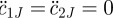 for all *I, J*.
— *M*_MAM_: all herds have the same probability of sending and receiving movements.

By implementing reduced versions of the full model, we aim to show what difference the included factors have on the dynamic and thereby indicate what aspects may be missed if these factors are excluded in modelling of spread of livestock disease via between-herd contacts. The full model is by no means a ‘true model’, but it is the most detailed description of the system.

To compare the dynamics predicted by the different models, we plot the mean number of infected individuals versus time from first infection. We are also interested in the variability in the initial stage of an outbreak and therefore also compare the final epidemic size as well as the distribution of currently infected and recovered herds at *t* = 1552, corresponding to the expected number of movements of four weeks. This was chosen because at this time, the number of infected herds was large enough to make relevant comparisons.

Assuming no interventions, the typical behaviour of an outbreak involves an initial increase of the number of infected herds because infections are generated faster than herds are recovering. As more herds are infected, the susceptible herds are depleted and eventually the rate of recovery is faster than the rate of new infections. When contacts between herds are not randomly distributed, we expect deviations from this pattern making the course of an outbreak less regular and to show fluctuations in the rate of spread during the initial increase as well as during the decrease [18]. Hence we are interested to measure and compare the amount of fluctuations in the rate of spread for each of the five models. To capture this effect of heterogeneity in contacts, we used a measure for marked fluctuations of the rate of spread. Yet, at a too fine time scale, we may expect frequent fluctuations in the rate of spread caused by the inherent randomness of the stochastic simulation model. To only include marked fluctuations, we identified the simulated number of infected herds at an extended time scale where ***τ*** = Δ*τ*, 2Δ*τ*, 3Δ*τ*, … using Δ*τ* = 776 (corresponding to the movements of two weeks) and defined *τ*_peak_ of each replicate as the time where the most herds are in the infected state at this scale. For *τ* < *τ*_peak_, corresponding to the phase of initial increase, we identified and counted the number of time steps satisfying *I*_*τ*_ < *I*_*τ*−Δ*τ*_ and for ***τ*** > *τ*_peak_ the number of time steps satisfying *I*_*τ*_ > *I*_*τ*−Δ*τ*_, where *I*_*τ*_ is the number of infected herds at time ***τ***. We define *ρ*_*x,m*_ as the proportion of time steps satisfying any of these two inequalities. This proportion gives an estimate of marked fluctuations of the rate of spread. We found that the choice of Δ*τ* = 776 was most suitable to capture marked changes in the number of infected herds rather than small changes due to individual herds becoming infected or recovered. At slower time scales the fluctuations evened out while at faster the fluctuations from the inherent randomness were still present. Such random events are also more apparent when there is a small number of infected herds [22], therefore, we only included steps were *I*_*τ*_ > 10.

## 6. Results and discussion

**Figure 2.**
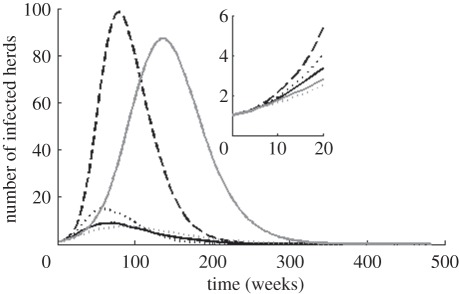
Mean number of infected herds versus time from first infection, given from 1000 simulations with each model, where *M*_full_ is the most detailed model and *M*_MAM_ assumes equal probability of infectious contacts. Other models listed in the legend refers to different simplifications of *M*_full_. Embedded axes show the same as main axes but only the 20 first weeks. Black: solid line, *M*_full_; dotted line, *M*_1_; dashed line, *M*_2_; grey: dotted line, *M*_3_; solid line, *M*_MAM_.

Figure 2 shows the mean number of infected herds at time after first infection. Removing the production-type structure, as is done in *M*_2_, has the highest impact when comparing the curves to the full model, *M*_full_. However, other factors are also essential as *M*_2_ differs from *M*_MAM_, most noticeably in the peak time of the curves. The maximum value for *M*_2_ occurs earlier, and there is initially a more rapid increase in the number of infected herds. Such dynamics is expected if some actors, in this case (generally) larger herds, are found to have more contacts [17]. Further, the final epidemic size is smaller for *M*_2_ than *M*_MAM_ (table 1) indicating that while there is initially on average a (slightly) higher rate of infections of *M*_2_, the reversed is found in later stages of the outbreak. Kiss *et al*. [23] conclude that heterogeneity in the number of contacts, which in *M*_2_ is introduced as an effect of herd size, may lead to a smaller final epidemic size. Also, *M*_2_ includes distance dependence in contact probability, which leads to local depletion of susceptibles. This effect is more prominent in later stages of an outbreak, and therefore more so in *M*_2_ than *M*_full_ as the resulting epidemic usually was larger.

**Table 1.** Summary statistics of number of infected herds.

	*M*_full_	*M*_1_	*M*_2_	*M*_3_	*M*_MAM_
final epidemic size^a^	79.0 (1, 1011)	100 (1, 1083)	611 (1, 1796)	86.5 (1, 1319)	787 (1, 2115)
infections > 1 (%)^b^	39.3	41.0	52.4	43.8	63.3
mean after four weeks^c^	1.23	1.24	1.26	1.26	1.22
variance after four weeks^d^	2.74	2.99	1.82	2.07	1.13

^a^Given by the mean and 2.5% and 97.5% percentiles (in brackets) as generated by simulations (1000 replicates) with each model.

^b^Percentages of simulations where at least one secondary infection was generated.

^c,d^The mean number of infected herds four weeks after initial infection. See text for details of the models.

The effects of between-herd distances and herd sizes are also shown more explicitly in figure 2 by the difference between *M*_*1*_ and *M*_3_, respectively, and the full model *M*_full_. Both show a slightly larger mean epidemic size (table 1), and because some herds act as superspreaders, the mean peak time of *M*_3_ is shifted right compared with the peak of *M*_full_.

The embedded axes in figure 2 show the mean differences in number of infected herds at the initial stages of the simulated outbreaks, but important features of the outbreak are neglected if only the mean is considered. Table 1 shows the pattern of the initial phase given by the mean and variance of the number of infected herds after four weeks as well as the proportion of replicates that result in at least one secondary infection before extinction. There is little difference between the means, but there is a large discrepancy in the variances. Hence, while *M*_MAM_ is a good predictor of the mean number of cases at the initial stage (there is little differences between *M*_MAM_ and *M*_full_ in the embedded axes of figure 2), it overlooks the possibility of rapid increase in the number of infected herds. Some production types, such as nucleus herds and multiplying herds, send many animals to other herds and while these types are rare, they may function as super spreaders and rapidly spread the disease to many other herds if they are infected at an early stage of an outbreak. The simulations are initiated with one randomly infected herd and most herds are of types that rarely send animals other than to slaughterhouses.

**Figure 3.**
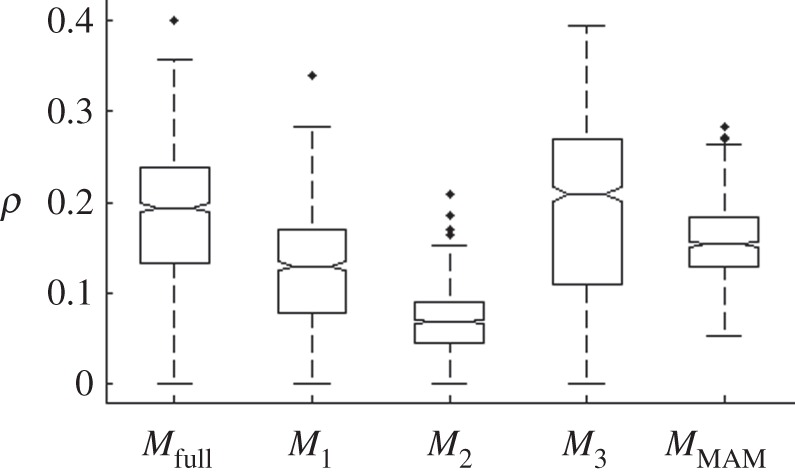
Boxplot of the stochastic measurement ***ρ*** from simulations with each model. Replicates with final epidemic size less than 10 herds were excluded. Notches show 95% confidence interval of the median, the box edges indicate 25th and 75th percentiles, respectively, and whiskers indicate the range of the distribution within 2.7 standard deviations. Outliers are indicated by plus symbol.

The production-type structure also causes unpredictable dynamics in the later stages of the outbreak scenario. The lowest median of *ρ* was found for *M*_2_, where the production-type structure was removed. With inclusion of the hierarchical production-type structure, the course of the outbreak depends highly on which herds are affected. Infections upwards in the pyramid are rare (e.g. from piglet producers to nucleus herds), but if they occur they may rapidly generate a large number of infections. Model *M*_1_, where distance dependence was removed, also showed a significantly lower *ρ* than *M*_full_ (shown by non-overlapping notches in figure 3). The spatial kernels estimated in Lindström *et al*. [5] were mainly leptokurtic, i.e. a fat tail describing long-distance contacts. These may spark new infections in areas where local depletion of susceptibles has not yet occurred, resulting in a new rise in the number of infections. Such dynamics were observed for instance in the UK 2001 outbreak of foot and mouth disease [24]. The estimates of *ρ* for *M*_3_, where herd-size effect was removed, showed a slightly higher, yet not significant, median than *M*_full_ (figure 3). We may however conclude that the herd-size effect reduces unpredictable dynamics because the median of *M*_MAM_ is significantly larger than both *M*_1_ and *M*_2_. If removal of herd size had no effect, we would expect that removal of all heterogeneities (as is done in *M*_MAM_) would result in more predictable dynamics than removal of just one of this factors removed in *M*_1_ or *M*_2_. To avoid the false impression that unpredictability implies low accuracy of the model, it needs to be stressed that the heterogeneous dynamics, here in particular caused by the production-type structure, is a realistic and important feature of disease spread modelling.

The aim of this study was to perform an in-depth analysis of animal movement heterogeneities and consequences for disease transmission dynamics. Rather than using expert opinions of contact patterns, which is the main means of parametrization in other simulation models, such as Interspread and Intercsf [25], our model is based on data from central databases of animal movements. In a previous paper [5], we describe in-depth how posterior distributions of parameters are estimated with Bayesian inference. Hence, parameter uncertainty is included in the simulation. However, the actual simulation set-up is very similar to those of Interspread and Intercsf, and we argue that the Bayesian approach where posteriors are estimated with MCMC may be incorporated into simulation models. In combination with expert opinions, the simulations can be adapted to specific diseases. As such, the model would most often also need to include other means of infections. We believe that the qualitative results regarding the effect of contact heterogeneities has a general validity but the effect of the considered contact heterogeneities may potentially be dwarfed or amplified by other infection pathways. For instance, other pathways are also expected to be more common at shorter distances and because of local depletion of susceptibles, the effect of distance dependence in the animal movement contacts would probably become more important.

In our simulation model, each replicate was initialized with the index herd picked randomly. A simulation model applied to a specific disease would also likely include deviations from this assumption. Arguments may be made that perhaps larger farms are more likely index herds because they have more contacts. However, Nöremark *et al*. [26] showed that farmers with larger herds were more aware of biosecurity issues, which might make these less likely to become index herds. Similarly, in a more applied model, a susceptible herd would generally not be deterministically infected if it receives animals from an infected herd but rather by some probability. Biosecurity would influence this probability for most diseases and a sufficient representation of the heterogeneous contact structure will likely be even more important in a simulation model where also the biosecurity is influenced by these heterogeneities. Further, we have also excluded within herd dynamics because we are trying to capture the effect of the contact heterogeneities rather than the dynamics of a specific disease. We argue that the qualitative results presented here regarding the importance of these heterogeneities ought to be valid also with inclusion of within herd dynamics. The dynamics of diseases that are rapidly infectious are most likely to resemble the prediction of our model, while diseases that are less infectious would result in fewer infected herds as well as a delay in the peak of the number of infected herds (i.e. in figure 2).

The approach of this study was to use a model that captures as much details of the contact structure as possible and by removing of heterogeneities one may test the effect of these. A slightly different approach would be to estimate the parameters of the reduced models separately. While this may seem more intuitive, it would make it more difficult to see the effect of the heterogeneous contact structure because herd size and production types are not independent. From a phenomenological point of view, the models could possibly behave similar but it would give less insight into the effect of the observed heterogeneities and make the results less relevant to other systems. It is however also important to point out that our results may not hold for all animal movement networks. For instance, in less-structured industries, such as sheep or most cattle production, production types may have far less importance. We suggest that similar analyses as the one presented here should be performed on other movement data in order to generate a more detailed and specific knowledge regarding the importance of heterogeneous contact structures. This will give deeper insight into what data need to be collected in order to make epidemiological predictions. Our results do point out the importance of correct data on production types in these databases and in general stresses that there is a need for continuous validation of the data on production type.

## 7. Conclusion

Of the factors included in the full model, the production-type structure is the factor most influential on the dynamics and hence the most important factor to obtain valid data for and include for modelling. Exclusion of this structure overestimates the final epidemic size but underestimates the variability of outbreaks, both in initial and later stages. However, contact heterogeneities due to herd sizes and between-herd distances also contribute substantially to the dynamics. Exclusion of the former leads to underestimation of the initial growth of the epidemic but slightly overestimate the final epidemic size. Also, if contact probabilities are modelled as independent of between-herd distances, the final epidemic size is somewhat overestimated. Exclusion of production-type differences in contact probabilities due to distance however underestimated the epidemic size in the analysed system. While the presented work only takes into account disease transmission via animal movements it still demonstrates that detailed and updated registers on the animal population in which a model is applied is essential. For a structured farming industry, such as the pig farming considered here, production types are particularly important. This may not be the most important factor in less-structured industries, but our result show that it primarily have to be considered and tested before applying modelling and risk assessment.
